# Immunohistochemical localization of VEGFR-2 in mouse mammary gland during reproductive cycle

**DOI:** 10.5455/javar.2021.h548

**Published:** 2021-11-01

**Authors:** Mohammad Saiful Islam, Mitsuharu Matsumoto

**Affiliations:** 1 Department of Anatomy, Histology & Physiology, Faculty of Animal Science & Veterinary Medicine, Sher-e-Bangla Agricultural University, Dhaka, Bangladesh; 2 Laboratory of Anatomy, Department of Basic Veterinary Sciences, Joint Faculty of Veterinary Medicine, Kagoshima University, Kagoshima, Japan

**Keywords:** Angiogenesis, mammary gland, immunohistochemistry, immunoblotting, VEGF, VEGFR-2

## Abstract

**Objective:**

The objective of this study was to obtain an insight into the role of the vascular endothelial growth factor (VEGF) in pregnancy-associated mammary epithelial development and angiogenesis. However, we examined the primary VEGF receptor (VEGFR-2) in the mouse mammary cycle.

**Materials and Methods:**

The virgin (10–12 weeks), days 10 and 18 of pregnancy (P-10 and P-18), days 0, 5, 10, and 20 of lactation (L-0, L-5, L-10, and L-20), and days 5 and 10 of post-weaning (W-5 and W-10) stage were all used in this study. Immunohistochemistry and Western blotting were carried out on mammary tissues taken from three mice at each stage.

**Results:**

VEGFR-2 was detected immunohistochemically in the cytoplasm of mammary epithelial and endothelial cells. Immunostaining for VEGFR-2 was consistently positive in mammary endothelial cells across all stages, whereas staining intensity in epithelial cells varied across the mammary cycle. Additionally, immunoblot analysis indicated a 220 kDa unique band of VEGFR-2 protein at all stages of the mammary cycle, with the maximum expression reported toward the end of pregnancy and gradually decreasing toward the end of lactation.

**Conclusion:**

In conclusion, the presence of VEGFR-2 in the mammary epithelium in addition to the endothelium suggests that VEGF plays an autocrine and paracrine role in the development, proliferation, and differentiation of the mammary epithelium during pregnancy.

## Introduction

The major factor in angiogenesis [1], vascular endothelial growth factor (VEGF), is involved in pregnancy-associated mammary growth, development, and functional differentiation [2,3]. The mammary parenchyma expands dramatically during pregnancy [4] and develops the distinctive lobuloalveolar structures that totally replace adipose tissues. It has been established that during pregnancy, mammary endothelial cell proliferation (angiogenesis) rises concurrently with mammary epithelial cell proliferation and that the mammary vasculature doubles as a result of sprouting angiogenesis [5]. At the end of pregnancy, intussusceptions further modify the mammary vasculature [6], and endothelial permeability peaks in response to increasing blood supply and milk production [5]. Previously, it was established that the mammary epithelium is the primary source of VEGF in pregnant and lactating mouse mammary glands [7], and inhibition studies of VEGF [8] further establish that mammary epithelium-derived VEGF is required for the growth and function of the mammary gland. 

The majority of VEGF’s biological effects are mediated through its receptors, VEGFR-1 (also known as Flt-1) and VEGFR-2 (also known as Flk-1) [1,9]. Additionally, neuropilin (NRP-1 and NRP-2) functions as a VEGF co-receptor [1]. It is generally documented that VEGFR-2 is the primary mediator of VEGF, whereas VEGFR-1 acts as a negative regulator of VEGFR-2 [1] and prevents endothelial cell proliferation from becoming uncontrolled. 

To date, research has demonstrated that VEGF/VEGFR-2 is expressed on a variety of non-endothelial cells in addition to endothelial cells [10,11], indicating its pleiotrophic effects. Only a limited amount of research has been conducted on the placement and expression of the VEGFR-2 receptor during the mammary reproductive cycle [2,3]. Although the expression and location of the VEGFR-2 transcript in the mouse mammary cycle have been previously characterized [3], the localization of the VEGFR-2 protein in the mouse mammary gland has not been described. The purpose of this study was to characterize VEGFR-2 expression in the mouse mammary gland during the reproductive cycle and to determine whether the changing pattern was associated with VEGF expression [7] and/or corresponded to the increased endothelial cell proliferation observed during pregnancy and lactation [6].

We explored the localization and expression of VEGFR-2 in the mouse mammary cycle to gain a better understanding of the VEGF system’s role in pregnancy-associated mammary angiogenesis and vascular permeability. Additionally, we evaluated blood vessel density throughout the mammary cycle.

## Materials and Methods

### Animals

Female Jcl-ICR mice were produced and maintained in our laboratory as a closed colony. They were housed in a climate-controlled and air-conditioned room (temperature: 23 ± 3°C, humidity: 60%, light–dark cycle: 12–12 h) and fed a commercial meal (Oriental Yeast Co., Tokyo, Japan) and tap water *ad libitum*. Throughout the trials, all animals were handled in accordance with the Institutional Animal Care and Use Committee of Kagoshima University [7,12]. Day 0 of pregnancy was defined as the day a vaginal plug was observed, while day 0 of nursing was defined as the day of parturition. Dams who were lactating were permitted to nurse their pups, which were removed shortly prior to sampling. The stage (10–12 weeks), days 10 and 18 of pregnancy (P-10 and P-18), days 0, 5, 10, and 20 of lactating (L-0, L-5, L-10, and L-20), and days 5 and 10 of post-weaning (W-5 and W-10) stages were all used in this study. Immunohistochemistry and Western blotting were carried out on mammary tissues taken from three mice at each stage. Each dam was normalized with her 8–10 pups during lactation and then removed from the young following the completion of the lactation period. 

### Tissue preparations

Mice were exsanguinated while anesthetized with a combination of ketamine and medetomidine. Immunohistochemistry was carried out on the right first abdomino-inguinal mammary gland immediately after it was excised and preserved in Zamboni solution. Left gland snaps were frozen in liquid nitrogen and stored at −80°C until further extraction. 

### Immunohistochemistry 

Following thorough washing in 0.1 M phosphate buffer (pH 7.4) at 4°C, the samples were routinely embedded in paraffin and sectioned at a thickness of 4 μm.. Immunostaining for VEGFR-2 and CD31 was carried out on consecutive serial sections. Endogenous peroxidase was quenched for 30 min with 3% H_2_O_2_ in methanol, and non-specific binding was blocked for 1 h at room temperature with 1% BSA/PBS. After that, the sections were treated overnight at 4°C with rabbit monoclonal anti-VEGFR-2 (Cell Signaling Technology, Danvers, MA; 1:500) and goat polyclonal anti-CD31 (Santa Cruz Biotechnology, Inc., CA; 1:1800). Secondary antibodies against VEGFR-2 and CD31 were anti-rabbit IgG and anti-goat IgG (Vector Laboratories, Burlingame, CA) diluted as 1:200. The antigens were seen using an Elite ABC kit (Vector Laboratories) and diaminobenzidine (DAB). Immunoreactions were halted and counterstained with Mayer’s hematoxylin. The appearance of a brown precipitate indicated positive reactions. Negative controls included incubation with matched IgG (Dakocytomation, Glostrup, Denmark) or PBS in place of the primary antibody.

The degree of VEGFR-2 immunostaining was semi-quantified at various phases using a graded scale: 0 indicates no staining; 1 indicates weakly positive staining; 2 indicates moderately positive staining; and 3 indicates substantially positive staining.

The number of CD-31-positive endothelial cells was counted at various periods of the mammary cycle and expressed as microblood vessel density (MVD). MVD was quantified using low magnification images of three randomly selected adjacent, non-overlapping fields of each segment and expressed as capillary density/mm^2^. 

### Western blotting

At 4°C, mammary tissues were homogenized for 30 min with continual shaking in Tris-buffered saline (TBS; pH 8.0) containing 1% Triton-X-100 and a protease inhibitor cocktail (Complete Mini; Roche, Mannheim, Germany). Following centrifugation (15,000 *g* for 30 min), supernatants were chilled prior to further analysis. 

The protein content was determined using the Pierce Biotechnology BCA Protein Assay kit (Rockford, IL) and corrected to 2 mg/ml using 5× non-reducing lane marker sample buffer (Pierce Biotechnology, Rockford, IL) 2-mercaptoethanol. After boiling for 5 min, the samples were incubated on ice. Following that, 7.5% SDS-PAGE was used to separate 20 gm protein from each homogenate using a Precision Plus protein standard (Bio-Rad, Hercules, CA) as a molecular weight marker. After electrophoresis, the proteins were transferred to PVDF membranes (Hybond-P; GE Healthcare Biosciences). After blocking overnight with 5% (w/v) skimmed milk in TBS (pH 8.0), the blot was probed with anti-VEGFR-2 (1:3000, rabbit monoclonal antibody, Cell Signaling Technology, Danvers, MA, United States of America) in blocking solution for 2 h at RT and then with horseradish peroxidase-conjugated donkey anti-rabbit antibody Chemiluminescence detection of antigen–antibody complexes was carried out according to the manufacturer’s instructions using the ECL detection kit (ECL-plus; GE Healthcare Biosciences). The intensity of the immunoreactive bands were assessed by scanning the photographic film and evaluating the generated image on a computer using National Institutes of Health (NIH) imaging software. To begin, we subtracted the background density from the density of each band. Following that, protein densitometric data were standardized to virgins. The mean integrated density for each band was calculated to determine the VEGFR-2 relative optical density (OD) units.

### Statistical analysis

The data from immunoblotting and immunohistochemistry densitometric analyses are provided as means standard error (SE). Analysis of variance was used for statistical analysis, and SPSS software was used (Version16.0; SPSS Inc., Chicago, IL). *p* < 0.05 was judged as statistically significant.

## Results

### VEGFR-2 Immunohistochemistry

**Figure 1.**
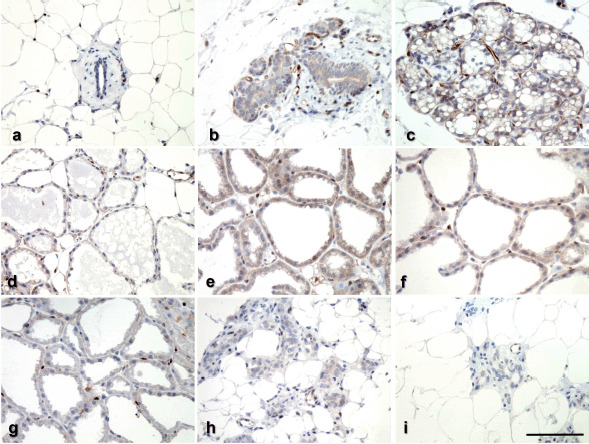
Immunohistochemical localization of VEGFR-2 among the various stages of mouse mammary gland development. Virgin (a); days 10 and 18 of pregnancy (b, c); days 0, 5, 10, and 20 of lactation (d, e, f, g); and days 5 and 10 post-weaning (h, i). Bar = 50 μm.

**Figure 2.**
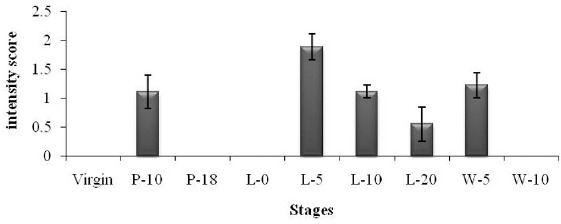
Change in VEGFR-2 immunoreactivity in the mammary epithelium of virgin, pregnant (P-10 and P-18), lactating (L-0, L-5, L-10, and L-20), and post-weaning (W-5 and W-10) mice. Each datum represents mean ± SE. Change in immunostaining intensity was significant (*p *< 0.01).

Immunostaining for VEGFR-2 was seen in mammary epithelial and endothelial cells and appeared to be predominantly in the cytoplasm, with some nuclear staining observed in the endothelium. As expected, the greatest immunostaining for VEGFR-2 was observed in mammary endothelial cells at all phases, serving as an internal positive control. In comparison, the intensity of VEGFR-2 staining in epithelial cells varied throughout the mammary cycle (Fig. 1), and thus was assessed in comparison to an internal control in all groups of animals, as shown in Fig. 2. The mammary parenchyma of virgin mice exhibited no response (Fig. 1a). The proliferating epithelium was slightly positive throughout early pregnancy (P-10), but became negative toward the end of pregnancy and parturition (Fig. 1b–d). At the commencement of lactation, the alveolar epithelium exhibited modest cytoplasmic reactivity (Fig. 1e), which persisted until the middle of lactation (L-5) (Fig. 1f), and then decreased significantly toward the end of lactation (L-20) (Fig. 1g). In post-weaned animals (W-5), partially collapsed alveoli exhibited a small reactivity (Fig. 1h), whereas nearly regressed epithelium (W-10) exhibited no reaction comparable to virgin mice (Fig. 1i). In negative control sections of mammary gland stained with normal rabbit serum, no immunolabeling was seen (data not shown).

### CD31 Immunohistochemistry

**Figure 3.**
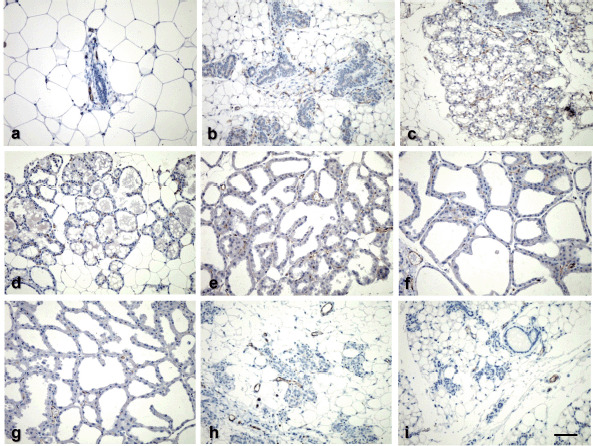
Immunohistochemical localization of CD31 among the various stages of mouse mammary gland development. Virgin (a); days 10 and 18 of pregnancy (b, c); days 0, 5, 10, and 20 of lactation (d–g); and days 5 and 10 post-weaning (h, i). Bar = 25 μm.

**Figure 4.**
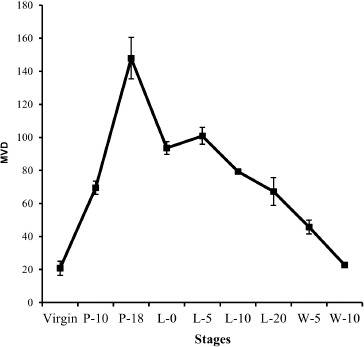
Changes of MVD in virgin, pregnant (P-10 and P-18), lactating (L-0, L-5, L-10, and L-20), and post-weaning (W-5 and W-10) mice. The endothelial cells were immunostained by anti-CD31 antibody and semiquantified. Each datum represents mean ± SE. Change in MVD among stages was significant (*p *< 0.01).

To determine whether vascular density changes during the mammary cycle, endothelial cells were immunostained with an anti-CD31 (also known as PECAM-1) antibody, a strong pan-endothelial cell marker. Early in pregnancy (P-10), an increase in endothelial cells resulted in the formation of a vascular network around the alveoli (Fig. 3b), and by the end of pregnancy (P-18), the lobuloalveolar unit is entirely encased by capillary loops (Fig. 3c). Quantitative data on vascular density demonstrated that MVD grows during pregnancy and reaches its peak at the end (Fig. 4). However, capillary density falls somewhat during lactation compared to pregnancy, which is likely due to larger alveoli distend the capillaries. Along with the epithelium, capillaries regress in post-weaned mice, as seen by the loss of pericytes (Fig. 3h and i) and MVD drops significantly, with totally regressed and reverted mammary glands exhibiting the lowest number of endothelial cells comparable to the virgin stage. 

### Immunoblotting

**Figure 5.**
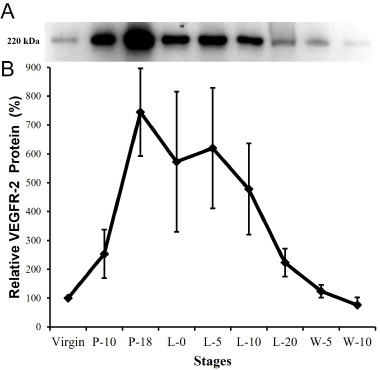
VEGFR-2 protein expression in the mouse mammary gland during the reproductive cycle. (A) Immunoblot analysis for VEGFR-2 is carried out at the virgin, pregnant (P-10 and P-18), lactation (L-0, L-5, L-10, and L-20) and post-weaning (W-5 and W-10) stages. Signal for VEGFR-2 is evident at 220 kDa. (B) Summary data for relative protein abundance of VEGFR-2 protein. The results are representative of three different experiments. Means ± SE are depicted for protein abundance expressed as OD of virgin mice. Change in VEGFR-2 protein among stages was significant (*p < *0.01).

VEGFR-2 antibody found a 220 kDa-specific band of VEGFR-2 at all stages of the mammary cycle. Figure 5 shows the changes in VEGFR-2 protein expression during the mammary cycle. In comparison to the virgin stage, the VEGFR-2 protein grew steadily during early pregnancy (P-10) and peaked at the end of pregnancy (P-18), before gradually decreasing near the end of lactation (L-20). Protein abundance fell significantly in post-weaned animals, reaching a minimum in totally regressed and reversed mammary glands (W-10), and was comparable to that reported in the pre-pregnant state. 

## Discussion

The VEGF has been discovered as a critical angiogenic, permeability, and survival factor that is largely expressed on endothelial cells via the VEGFR-2 receptor. However, mounting evidence suggests that the VEGF has a far broader and pleiotrophic role, with findings indicating VEGFR-2 expression on a range of non-endothelial cells [10,13]. To our knowledge, this is the first study to use immunohistochemistry and immunoblotting to evaluate the localization and expression of VEGFR-2 protein in virgin, pregnant, nursing, and involuted mouse mammary glands. 

Immunohistochemical analysis revealed that the VEGFR-2 protein is expressed in the mammary epithelium and the vascular endothelium. During pregnancy, the VEGFR-2 is highly expressed in the endothelium, but has a low level of reactivity in proliferating epithelial cells. VEGFR-2 expression was moderate in the mammary alveoli during the early stages of lactation but reduced toward the end of lactation. The current study found that VEGFR-2 is predominantly expressed by mammary endothelial cells during pregnancy, adding to the growing body of evidence and suggesting that the VEGF/VEGFR-2 system plays an angiogenic role [8] in the pregnancy-induced mammary gland. Additionally, the presence of VEGFR-2 in breast epithelial cells throughout early pregnancy and lactation indicates that epithelial cells contribute to some extent to VEGFR-2 expression. The current findings, which corroborate our previous findings [7], indicate that mammary epitheliums are not only a source of VEGF, but also a target of this multifunctional substance, and also support the concept that this cytokine acts as an autocrine and paracrine factor [11,13] in pregnancy-associated mammary epithelial growth, proliferation, and differentiation. 

We describe the VEGFR-2 protein in the mouse mammary gland for the first time using immunoblotting. In agreement with previously published research [11,13], we also observed the VEGFR-2-specific 220 kDa band in endothelial and non-endothelial cells. Densitometric analysis of the immunoblot data revealed that VEGFR-2 expression is differentially expressed during the mouse mammary cycle. Following pregnancy, the VEGFR-2 protein level grew gradually and reached a maximum toward the end of pregnancy. Throughout lactation, VEGFR-2 expression fell progressively until the end of lactation, when it dramatically plummeted in involuted mice (Fig. 5B). Our immunoblot and semiquantitative findings reveal that endothelial cells are the primary source of VEGFR-2 during pregnancy, when endothelial proliferation (angiogenesis) is at its peak (as evaluated by MVD). At the end of pregnancy, sprouting angiogenesis ceases and existing capillaries undergo additional remodeling (intussception) [6]. VEGFR-2 expression may be related with vascular remodeling (intussusceptions), permeability, and survival during early lactation (L-5, L-10). Additionally, earlier research has established that VEGF acts as an autocrine survival factor in various types of epithelium [11,13,14]. Our current discovery that VEGF has a low level of reactivity in mammary epithelial cells during early to mid-lactation suggests that VEGF may block early apoptosis and act as a survival factor for breastfeeding epithelial cells. Additionally, the presence of VEGFR-2 in early lactating mammary epithelial cells suggests that lactating mammary epithelial cells may directly regulate their own blood supply and vascular permeability.

Additionally, we studied changes in vascular pattern between stages of the mammary cycle using CD31 antibodies to highlight endothelial cells. The quantification of mammary endothelial cells demonstrated that MVD density grows gradually during pregnancy and is highly expressed toward the end of pregnancy, progressively decreases throughout advanced lactation, and then rapidly decreases in post-weaned mice. Consistent with our findings, Pepper et al. [3] likewise discovered the highest capillary density at the end of pregnancy, which gradually declined during lactation, and the lowest capillary density in the involuted mammary gland. The MVD data further corroborate our VEGFR-2 immunoblot data, indicating that the mammary gland vasculature experiences substantial angiogenesis during pregnancy. Previously, it was observed that pregnancy and lactation have a significant effect on the mouse mammary vasculature [5]. Ultrastructural examination of the mammary gland [6] demonstrates that the mammary vasculature undergoes considerable angiogenesis (formation of capillary sprouts) at the end of pregnancy and thereafter vanishes. In early lactation, non-proliferative angiogenesis (intussusceptions) remodels the mammary vasculature [6]. Taken together, we hypothesized that angiogenesis during pregnancy could be regulated directly by mammary epithelial cells, as the mammary epithelium is the primary source of VEGF during pregnancy [3,7]. 

Previous investigations in the rodent mammary cycle [2,3] have demonstrated that VEGFR-2 mRNA expression fluctuates during developmental stages with contradictory results. Pepper et al. [3] demonstrated that VEGFR-2 mRNA increased considerably during pregnancy, peaked during early lactation, and then steadily reduced in post-weaned mice using northern blot, whereas Hovey et al. [2] discovered the lowest level around parturition. Apart from the methodological differences, the discrepancy in mRNA quantification between the two is unclear. Consistent with our previous findings [7], the VEGF receptor protein undergoes similar changes, demonstrating that its ligand regulates VEGFR-2 expression during the mammary gland reproductive cycle.

The immunohistochemical data for VEGFR-2 given in this work differ from Hovey et al.’s earlier report on the distribution of VEFR-2 mRNA in the mouse mammary gland [2]. *In situ* hybridization was used to locate the VEGFR-2 transcript in adipose tissues and stromal fibroblast cells in this work [2]. In comparison, we observed the expression predominantly in the mammary epithelium and blood vessels. Our result, however, is consistent with earlier human investigations [14,15]. The disagreement could be related to methodological differences, and it is most likely that Hovey et al. [2] concentrated on stromal cells as the primary location of VEGF/VEGFR-2 expression rather than the mammary epithelium.

Nitric oxide (NO), another important mediator of angiogenesis and vascular permeability that is released from endothelial cells [16,17] upon activation of the endothelial nitric oxide synthase (eNOS), is required for VEGF’s pro-angiogenic effects. Numerous investigations have demonstrated that NO plays a significant role in VEGF-induced vascular permeability and angiogenesis [18–21]. We previously described the expression of eNOS during the mouse mammary gland reproductive cycle [10]. Given that VEGFR-2 is responsible for the majority of VEGF’s functional and biochemical effects in endothelial cells, including NO release [18], we hypothesized that pregnancy-induced angiogenesis and vascular permeability could be achieved through a synergistic interaction of VEGF/VEGFR-2/NO [19] in pregnant and lactating mammary glands. 

Estrogen and progesterone are now well recognized as being absolutely necessary for mammary epithelial proliferation and differentiation [9,22,23]. Recent research studies have established estrogen’s angiogenic properties in the female reproductive tract [22,23]. Estrogen appears to generate angiogenesis in the pregnant mammary gland [Dabrosin] by generating the major angiogenesis factor VEGF/VEGFR-2 and regulating VEGF-induced angiogenesis in the pregnant mammary gland.

## Conclusion

The current work established the localization and expression of VEGFR-2 in both mammary epithelium and endothelium at distinct stages of the mouse mammary cycle. In agreement with our previous findings, the current data strongly suggest that the VEGF ligand–receptor system controls pregnancy-induced lactation angiogenesis and vascular permeability in an autocrine–paracrine manner. Additional research is required to explain the ligand–precise receptor’s role in mammary epithelial development, proliferation, survival, and apoptosis. 

## List of abbreviations

BSA/PBS, bovine serum albumin/ phosphate buffer saline; BCA, bicinchoninic acid; PVDF, polyvinylidene fluoride; PECAM, platelet endothelial cell adhesion molecule 
